# 27-hydroxycholesterol and DNA damage repair: implication in prostate cancer

**DOI:** 10.3389/fonc.2023.1251297

**Published:** 2023-12-21

**Authors:** Gloria Cecilia Galvan, Nadine A. Friedrich, Sanjay Das, James P. Daniels, Sara Pollan, Shweta Dambal, Ryusuke Suzuki, Sergio E. Sanders, Sungyong You, Hisashi Tanaka, Yeon-Joo Lee, Wei Yuan, Johann S. de Bono, Irina Vasilevskaya, Karen E. Knudsen, Michael R. Freeman, Stephen J. Freedland

**Affiliations:** ^1^ Department of Urology, Samuel Oschin Comprehensive Cancer Institute, Cedars-Sinai Medical Center, Los Angeles, CA, United States; ^2^ Department of Urology, University of California, Los Angeles, Los Angeles, CA, United States; ^3^ Urology Section, Department of Surgery, Veterans Affairs Health Care System, Durham, NC, United States; ^4^ Department of Pathology, Duke University School of Medicine, Durham, NC, United States; ^5^ Department of Surgery, Cedars-Sinai Medical Center, Los Angeles, CA, United States; ^6^ Department of Biomedical Sciences, Cedars-Sinai Medical Center, Los Angeles, CA, United States; ^7^ Cancer Biomarkers Team, Division of Clinical Studies, The Institute of Cancer Research, London, United Kingdom; ^8^ Prostate Cancer Targeted Therapy Group and Drug Development Unit, The Royal Marsden NHS Foundation Trust, London, United Kingdom; ^9^ Department of Cancer Biology at Thomas Jefferson University, Philadelphia, PA, United States

**Keywords:** 27-hydroxycholesterol (27HC), prostate cancer, hydroxycholesterol, LNCaP (prostate cancer cell), CYP27A1, DU145 (prostate) cancer cell line

## Abstract

**Introduction:**

We previously reported that cholesterol homeostasis in prostate cancer (PC) is regulated by 27-hydroxycholesterol (27HC) and that CYP27A1, the enzyme that converts cholesterol to 27HC, is frequently lost in PCs. We observed that restoring the CYP27A1/27HC axis inhibited PC growth. In this study, we investigated the mechanism of 27HC-mediated anti-PC effects.

**Methods:**

We employed in vitro models and human transcriptomics data to investigate 27HC mechanism of action in PC. LNCaP (AR+) and DU145 (AR-) cells were treated with 27HC or vehicle. Transcriptome profiling was performed using the Affymetrix GeneChip™ microarray system. Differential expression was determined, and gene set enrichment analysis was done using the GSEA software with hallmark gene sets from MSigDB. Key changes were validated at mRNA and protein levels. Human PC transcriptomes from six datasets were analyzed to determine the correlation between CYP27A1 and DNA repair gene expression signatures. DNA damage was assessed via comet assays.

**Results:**

Transcriptome analysis revealed 27HC treatment downregulated Hallmark pathways related to DNA damage repair, decreased expression of FEN1 and RAD51, and induced “BRCAness” by downregulating genes involved in homologous recombination regulation in LNCaP cells. Consistently, we found a correlation between higher CYP27A1 expression (i.e., higher intracellular 27HC) and decreased expression of DNA repair gene signatures in castration-sensitive PC (CSPC) in human PC datasets. However, such correlation was less clear in metastatic castration-resistant PC (mCRPC). 27HC increased expression of DNA damage repair markers in PC cells, notably in AR+ cells, but no consistent effects in AR- cells and decreased expression in non-neoplastic prostate epithelial cells. While testing the clinical implications of this, we noted that 27HC treatment increased DNA damage in LNCaP cells via comet assays. Effects were reversible by adding back cholesterol, but not androgens. Finally, in combination with olaparib, a PARP inhibitor, we showed additive DNA damage effects.

**Discussion:**

These results suggest 27HC induces “BRCAness”, a functional state thought to increase sensitivity to PARP inhibitors, and leads to increased DNA damage, especially in CSPC. Given the emerging appreciation that defective DNA damage repair can drive PC growth, future studies are needed to test whether 27HC creates a synthetic lethality to PARP inhibitors and DNA damaging agents in CSPC.

## Introduction

Prostate cancer (PC) remains the most common non-cutaneous cancer and the second leading cause of cancer mortality amongst men in the United States. In 2023, it is estimated that 288,300 men will receive a diagnosis of PC, while approximately 34,700 lives will be lost to this disease (1). Notably, modifiable risk factors, especially dietary and lifestyle factors, are hypothesized to play a significant role in the prevention of PC (2, 3). Among these factors, cholesterol has been implicated in the development of PC for nearly a century (4). Recent studies have further demonstrated a link between cholesterol and an increased risk of PC incidence, as well as the development of aggressive tumors (5–8). Consequently, therapies aimed at reducing cholesterol bioavailability have been proposed as potential options for both prevention and/or treatment of PC (9, 10). Hydroxymethylglutaryl-CoA (HMG-CoA) reductase inhibitors, commonly known as statins, have shown promise in various population and retrospective studies showing an association between statin use and reduced risk of advanced/fatal PC, improved survival rates, and even reduced biochemical recurrence-free survival in men with PC after radical prostatectomy (11, 12). However, statins predominantly block cholesterol production in the liver and thus lower serum cholesterol levels. As such, tumors are potentially able to overcome this effect by increasing cholesterol uptake to maintain high intracellular cholesterol levels (13). Therefore, a better understanding of the impact of changing *intracellular* cholesterol levels is needed using tools beyond statins given that how cholesterol promotes aggressive PCs remains as an important unanswered question.

Of 176 cholesterol homeostasis genes, our group previously identified CYP27A1 as the only gene whose expression level was significantly associated with T-stage, Gleason score at diagnosis, and presence of lymph node metastasis in PC (14, 15). CYP27A1 encodes sterol 27-hydroxylase, a cytochrome P450 oxidase that converts cholesterol into 27-hydroxycholesterol (27HC). Via a feedback loop that activates transcription factors liver-X-receptors (LXRs) alpha and beta and represses sterol regulatory element-binding protein 2 (SREBP2), 27HC senses overproduction of cholesterol, reverses cholesterol transport out of cells and promotes its excretion from the cell, effectively protecting cells from excess cholesterol accumulation. In previous studies, we also found that CYP27A1 expression in PC tissue was lower than benign prostate tissue. Importantly, we discovered that restoration of CYP27A1 or treatment with 27HC, the metabolic endproduct of CYP27A1 activity, attenuates PC growh *in vitro* and *in vivo (* 14 *).*

In terms of the mechanism of 27HC activity, we recently showed that 27HC treatment blocked IL-6/JAK/STAT signaling, which may contribute to its anti-PC activity (15). Herein, we sought to identify other potential mechanisms of 27HC-mediated anti-PC effects by transcriptomic analysis of *in vitro* PC models. Results were confirmed using large scale human transcriptomic data along with biochemical and molecular analyses of cell models. We hypothesized that 27HC affects PC through multiple mechanisms beyond cholesterol inhibition including as yet poorly studied pathways that may interact with cholesterol homeostasis.

## Materials and methods

### Cell culture

Cell lines were purchased from ATCC (Manassas, VA). LNCaP (AR+, CYP27A1-) and DU145 (AR-, CYP27A1+) were cultured in Roswell Park Memorial Institute Medium (RPMI) or Dulbecco’s Modified Eagle Medium (DMEM), respectively, plus 10% fetal bovine serum (FBS), as previously reported (14). Non-neoplastic prostate epithelial cells, RWPE-1 (16), were kindly provided by the Freeman lab and grown in Keratinocyte Serum-Free Medium (K-SFM) plus growth-factor kit (Gibco 17005-042, Thermo Fisher Scientific, Waltham, MA). 27HC was purchased from Enzo Clinical Labs (Farmingdale, NY) and resuspended in DMSO as described previously (15). Roscovitine was purchased from Selleck Chemicals LLC (Houston, TX).

### Gene expression analysis

LNCaP and DU145 cells were treated with 27HC (10µM) or vehicle control (DMSO/ethanol) in triplicate for 48 hours. RNA extraction, cDNA amplification and microarray hybridization were performed at UCLA Technology Center for Genomics and Bioinformatics. Transcriptome profiling was done with Affymetrix GeneChip™ Human Genome U133 Plus 2.0 Array assay (Thermo Fisher Scientific). Data quality control and probe set normalization were done using Affymetrix Power Tools package and the single-channel array normalization (SCAN) algorithm, respectively (17, 18).

Differentially expressed genes (DEGs) between 27HC treated and control groups were determined using an integrated hypothesis testing method. Briefly, three p-values were computed from t-test, rank-sum test and median difference test. The p-values were combined into an overall p-value using Stouffer’s method. We then performed multiple testing correction using Storey’s method (19). Genes with a false discovery rate (FDR) less than 0.05 and absolute log2-median-ratio greater than the 95-percentile value from the null distribution of log2-median-ratio, which is based on 1,000 random permutation of samples. Gene set enrichment analysis was done using the GSEA software (20) with hallmark gene sets from Molecular Signature Database (MSigDB) (21). We used weighted Z-score method (22) to compute gene scores using median-centered and normalized gene expression values.

### Correlation analysis

We investigated the association between CYP27A1 (enzyme that modulates 27HC biosynthesis) and DNA repair genes. We used previously published lists of genes associated with homologous recombination (HR) to represent “BRCAness” (23) and with global human DNA repair (24). We then analyzed the expression of those genes in our microarray data from 27HC-treated PC cells. As reported by others, the list of 10 homologous recombination associated genes involved in “BRCAness” included: *CHEK1, BRCA1, EXO1, BLM, RMI1, RAD54L, RAD51, LIG1, XRCC3*, *RMI2* and *RPA1* (23). The DNA repair gene list was based on a previously published PC signature (24) and modified to include only the genes present in our dataset. Gene lists are included in **Tables S1, S2** of **Supplementary Materials**.

To study this association, CYP27A1 with HR and DNA repair genes, in human PC, we analyzed their expression using the signatures described above and the Hallmark DNA Repair Gene Set from MSigDB (21) in datasets from human PC. First, we used the public online tool Prostate Cancer Transcriptome Atlas (PCTA; thepcta.org), which includes data from 1,321 clinical specimens from 38 PC cohorts, mostly primary localized tumors. Analyses were stratified by Gleason score (GS<7, GS=7, GS>7) and data were shown separately for those samples from men with metastatic castration-resistant PC (mCRPC), when data were available. Computed enrichment signatures are shown as z-scores. The correlation coefficients between gene signature score and CYP27A1 expression were assessed via Spearman’s method and its significance level was tested with two-tailed test. Similar analysis was done in The Cancer Genome Atlas (TCGA) dataset using the PCTA web-interface. We also evaluated this association in human transcriptomes (n=2,000) from the Decipher Genomics Resource Information Database (GRID) registry (25) (NCT02609269) and from the Durham Veteran Affairs Health Care System (DVAHCS) cohort (n=554), which includes men treated for PC with radical prostatectomy between 1989-2016 at the Durham Veteran Affairs Health Center System in Durham, NC (26). In addition, we analyzed two independent mCRPC cohorts: SU2C-PCF (27) (n=159) and RMH (28) (n=98), which include tumor biopsy bulk cell RNAseq data.

### qRT-PCR

For validation of the microarray results, we determined expression of RAD51 and FEN1, two canonical genes involved in DNA repair due to their assistance in repair of double-stranded breaks in PC cells by qRT-PCR. LNCaP and DU145 cells were treated with 27HC (10µM) or vehicle control for 48 hours. When treated with roscovitine, LNCaP and DU145 cells were pre-treated with roscovitine (5µg/ml) and 24 hours later treated with 27HC (10µM) for 48 hours. After treatment, cells were rinsed with 1X PBS solution. RNA isolation was done by Trizol (Life technologies, Carlsbad, CA, USA). RNA was cleaned by sodium acetate. RNA concentration was determined by a Nanodrop spectrophotometer (Thermo Fisher Scientific). Reverse transcription was done with iScript cDNA synthesis kit (Bio-Rad Laboratories, Hercules, CA, USA). A total of 10 ng of cDNA per reaction combined with Superscript SYBR green (Bio-Rad Laboratories) were run on the ABI Viia7 RT-PCR system (Thermo Fisher Scientific). mRNA levels were normalized to housekeeping genes (GAPDH or Actin). Relative mRNA levels were determined using the delta delta CT method. Primer sequences are listed in **Table S3** of **Supplementary Materials**. Assays were run in triplicate with all results contributing to the statistical analysis. Two-sample t-test was used to compare groups. Statistical significance was defined by a p-value<0.05.

### Western blotting

After 27HC or control treatment of LNCaP, DU45 and RWPE-1, cells were fixed with 10% trichloroacetic acid for 30 min on ice, and the total cell lysates were prepared with LDS Sample Buffer (Thermo Fisher Scientific). Western blotting was performed with NuPAGE protein electrophoresis system (Thermo Fisher Scientific). Primary antibodies included rabbit anti-Rad51 (1:500, sc-8349, Santa Cruz Biotechnology, Dallas, TX, USA), rabbit anti-FEN1 (1:1000, Cell Signaling Technology, Danvers, MA, USA), mouse anti-phospho-Histone H2A.X (Ser139, 1:1000, 05-636-I, Millipore Sigma, St. Louis MO, USA), rabbit anti-phospho-KAP1 (1:1000, Bethyl Laboratories, Thermo Fisher Scientific), rabbit anti-phospho-RPA32 (1:1000, Bethyl Laboratories Thermo Fisher Scientific) and mouse β-actin (1:1000, Santa Cruz Biotechnology). Ponceau S staining solution (Thermo Fisher Scientific) was used to evaluate transfer efficiency and for total protein normalization. Bands were visualized with Immun-Star AP Chemiluminescence Kits (Bio-Rad Laboratories) and were detected with ChemiDoc Touch Imaging System (Bio-Rad Laboratories) and normalized to loading controls (β-actin/Ponceau S) first and then to each control group (0 µM). Results were run in triplicate.

### Comet assay

LNCaP and DU145 cells were treated as mentioned above in complete medium (10% FBS). The comet assay (OxiSelect™ Comet Assay Kit, Cell Biolabs Inc, San Diego, CA) was performed in alkaline conditions for evaluation of cellular DNA damage and was conducted under dim light to avoid ultraviolet damage to cell samples. An agarose base layer was created in each individual slide well and allowed to solidify. The cell samples were combined with the Comet Agarose at a 1:10 ratio and 75uL of this mixture was then applied to the top of each base layer per well and allowed to solidify at 4°C. Slides were incubated in lysis buffer for 1 hour, then alkaline solution for 30 min at 4°C in the dark. The slides were electrophoresed for 30 min at 24 volts, maintaining a current setting of 300mA in cold alkaline electrophoresis solution. Slides were transferred to cold DI H_2_O and washed twice, before a 5 min incubation in cold 70% ethanol. Slides were then allowed to air dry overnight. Once slides are completely dry, 100uL of diluted Vista Green DNA Dye was added to each well and allowed to incubate at room temperature for 15 min in the dark. After imaging, comet tails were quantified with OpenComet v1.3.1, an open-source software tool providing automated analysis of comet assay images (29).

## Results

### 27-hydroxycholesterol treatment suppresses expression of DNA damage repair genes in PC cells *in vitro*

**Figure 1:**
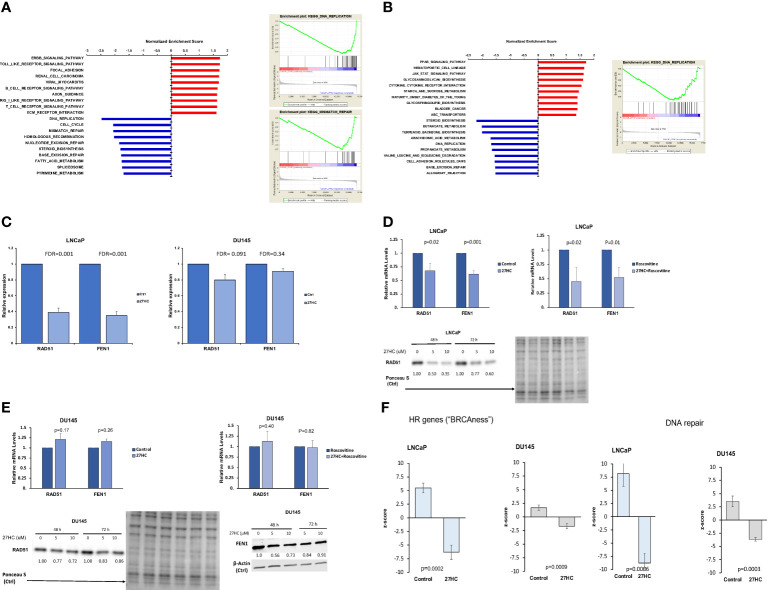
27HC treatment of PC cells suppresses genes involved in DNA damage repair. **(A)** Top 10 pathways enriched by 27HC in LNCaP cells. **(B)** Top 10 pathways enriched by 27HC in DU145 cells. **(C)** FEN1 and RAD51 gene expression in microarray analysis. Data show fold change of 27HC vs control. **(D, E)**. qRT-PCR validation of FEN1 and RAD51 expression in LNCaP and DU145 cells treated with 27HC (10µM) for 48 hours, with and without roscovitine (5µg/ml). Data show mean and standard deviation. Protein levels of RAD51 and FEN1 after 27HC treatment. Quantification shows relative expression to loading controls. **(F)** Effect of 27HC on “BRCAness”, shown by regulation of expression of HR related genes, and DNA repair gene expression in microarray analysis.

Gene expression analysis revealed that 27HC inhibited expression of multiple DNA damage response pathways in PC cells. In LNCaP cells, out of the top 10 27HC downregulated pathways, six were related to DNA repair and damage response, including DNA replication, cell cycle, mismatch repair, homologous recombination, nucleotide excision repair, and base excision repair hallmark pathways (**Figure 1A**). Meanwhile, in DU145 cells, while the link was not as robust, two of the top 10 downregulated pathways were related to DNA damage response, including DNA replication and base excision repair hallmark pathways (**Figure 1B**). From the microarray, when specifically looking at two canonical DNA repair pathway genes, RAD51, a key recombinase involved in homology-directed DNA repair, and FEN1, a flap endonuclease involved in DNA repair and replication, it was noted that 27HC treatment downregulated gene expression of both genes compared to control in LNCaP but not in DU145 (**Figure 1C**). When validating expression of RAD51 and FEN1 by qRT-PCR, we observed significantly lower mRNA levels in 27HC treatment vs. control in LNCaP cells (**Figure 1D**, RAD51 p=0.02, FEN1 p=0.001), but not in DU145 cells (**Figure 1E**, RAD51 p=0.17, FEN1 p=0.26). Given our previous knowledge of 27HC effects on cell growth inhibition and suppression of cell cycle gene expression (14) (as shown in **Figure 1A**), we tested if decreased expression of DNA damage response genes was mediated via cell cycle suppression. To address this, we treated PC cells with roscovitine, a CDK2/CDK1 inhibitor that leads to cell cycle arrest in late G1 phase (30). The qRT-PCR results show that even when all cells were treated with roscovitine, co-treatment with 27HC significantly lowered RAD51 and FEN1 mRNA levels compared to roscovitine treatment alone in LNCaP cells (**Figure 1D**, RAD51 p=0.02, FEN1 p=0.01). However, no inhibition of RAD51 and FEN1 was seen in DU145 cells treated with 27HC plus roscovitine (**Figure 1E**, RAD51 p=0.40, FEN1 p=0.82). These data suggest that 27HC inhibits expression of DNA repair genes in LNCaP cells independent of cell cycle effects. Downregulation of RAD51 by 27HC was confirmed by Western blot in LNCaP cells after 27HC treatment (5 and 10 µM) at 48 and 72 hours (**Figure 1D**) whereas in DU145 cells there was limited downregulation of RAD51 and FEN1 (**Figure 1E**). Additionally, using previously published gene signatures to interrogate the microarray data, we found 27HC treatment significantly downregulated genes involved in DNA repair (LNCaP p=0.0006, DU145 p=0.0003) and in homologous recombination leading to an induction of “BRCAness” (LNCaP p=0.0002, DU145 p=0.0009) (**Figure 1F**). These findings suggest 27HC treatment significantly downregulated genes involved in DNA repair and induced “BRCAness” across both cell lines and thus may make cells vulnerable to clinically available agents that cause DNA damage.

### Expression of CYP27A1 is associated with expression of DNA repair and “BRCAness” related genes in human PC

**Figure 2:**
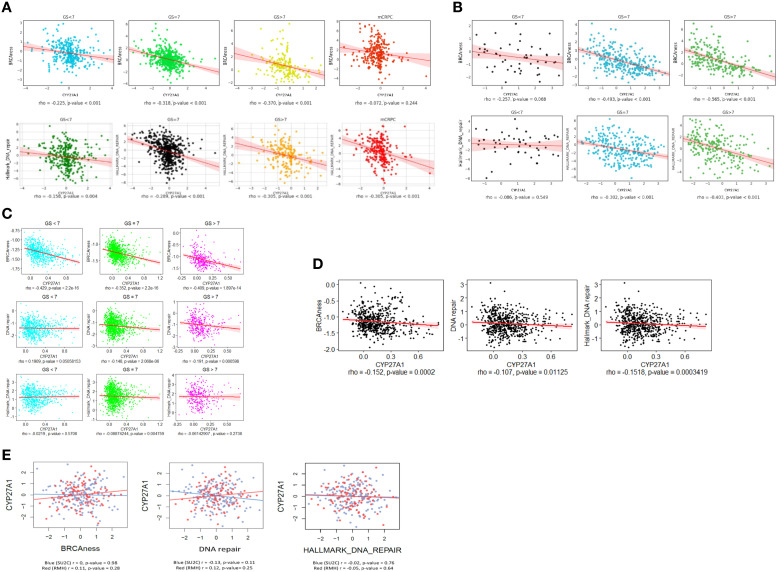
Correlation of CYP27A1 expression with DNA damage repair and “BRCAness” gene signatures in human PC. Plots show gene expression profiles from different transcriptome datasets and computed enrichment of signatures in each patient using the z-score method. P-values were calculated from Spearman’s correlation among z-scores between gene signatures and CYP27A1 expression for the following cohorts: **(A)** PCTA, **(B)** TCGA, **(C)** GRID, **(D)** DVAHCS and **(E)** SU2C and RMH. For PCTA, TCGA and GRID datasets, data is shown by disease course.

To determine if CYP27A1 expression is associated with expression of genes associated with DNA repair and “BRCAness” in human PC, we tested representative gene signatures in different human PC transcriptome datasets (PCTA, TCGA, GRID, DVAHCS, SU2C and RMH). Across all castrate-sensitive PC (CSPC) datasets (PCTA, TCGA, GRID, and DVAHCS), there were generally negative associations between expression of CYP27A1 and DNA repair genes (i.e., high CYP27A1 expression suggesting high intracellular 27HC, correlated with lower DNA repair pathway expression) (**Figure 2**). Although these associations often were highly statistically significant, some failed to reach statistical significance. When primary tumors were stratified by Gleason sum, associations were weakest in Gleason <7 tumors in 5 of 6 comparisons. Moreover, across the PCTA, TCGA, GRID and DVAHCS datasets and all Gleason scores, in primary tumors, associations of CYP27A1 expression with “BRCAness” signature were stronger compared to those between CYP27A1 and the Hallmark DNA repair pathway (**Figures 2A, D**). Finally, in in mCRPC samples of the PCTA dataset, associations only reached statistical significance for the Hallmark DNA repair pathway (**Figure 2A**). Given the mixed results in mCRPC vs. robust associations seen in primary tumors (especially higher-grade localized tumors), we explored additional cohorts with mCRPC. Specifically, we looked at the RMH and SU2C datasets, comprised of mCRPC cases, and found no correlation between expression of CYP27A1 and DNA repair and “BRCAness” gene signatures (**Figure 2E**). These findings suggest that in human primary CSPC samples, higher CYP27A1 and presumably higher intracellular 27HC is associated with decreased expression of DNA repair genes and these associations appear to be stronger in higher grade primary tumors. Alternatively, in mCRPC patients, results are largely null. As such, these findings validate the translational nature of our preclinical findings from the microarray including the fact that results for DNA repair expression downregulation were stronger in the hormone-sensitive cell line LNCaP vs. the androgen-independent cell line DU145 (**Figure 1**).

### 27HC treatment induced expression of DNA damage markers in PC cells but not in non-neoplastic prostate epithelial cells

**Figure 3:**
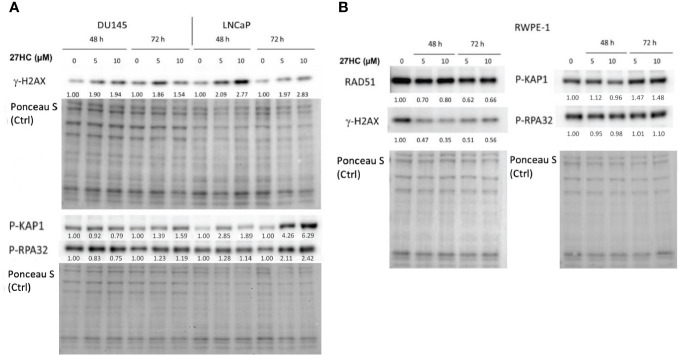
Effects of 27HC on DNA damage on PC and non-neoplastic prostate epithelial cells. Western blot and quantification of RAD51, gamma-H2AX, phospho-KAP1 and phospho-RPA32 in **(A)** DU145, LNCaP, and **(B)** RWPE-1 cells after treatment with 5µM or 10µM 27HC for 48 and 72 hours.

Given that we showed that 27HC decreased expression of DNA repair pathway genes, we next asked whether 27HC could induce DNA damage. To test this, we treated LNCaP and DU145 cells with 27HC (5 µM or 10 µM) for 48 and 72 hours (**Figure 3**) and measured protein level of general DNA damage marker γ-H2AX. 27HC treatment strongly induced γ-H2AX in LNCaP and to a lesser extent in DU145. We next measured pathway-specific DNA damage markers, p-KAP1 (DNA double strand break) and p-RPA32 (stalled replication forks). These markers also increased in LNCaP while no consistent results were seen in DU145 cells. However, we did not observe the same effect in RWPE-1 cells, wherein gamma-H2AX levels actually decreased with 27HC treatment. These findings suggest 27HC treatment selectively leads to DNA damage in PC cells but not in non-neoplastic prostate epithelial cells, with stronger effects in hormone sensitive LNCaP cells vs. androgen independent DU145 cells.

### Comet assay shows 27HC increased DNA damage in LNCaP cells and this was reversible by cholesterol and not androgens

**Figure 4:**
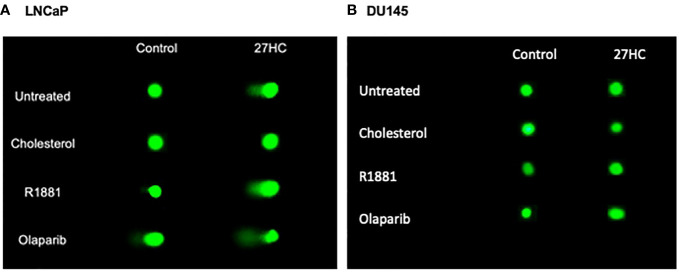
Comet assay on 27HC-treated LNCaP and DU145 cells plus R1881, cholesterol or olaparib. **(A)** LNCaP cells. **(B)** DU145 cells.

**Figure 5:**
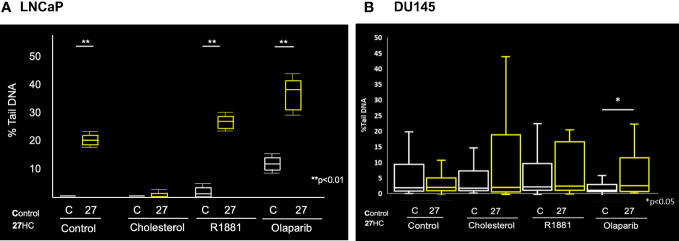
Quantification of comet assay results showing boxplots of percent tail DNA. **(A)** LNCaP cells. **(B)** DU145 cells.

Given above we found that 27HC could induce expression of DNA damage markers, we next sought to validate these findings using a different measure of DNA damage. Thus, we used a comet assay, which uses gel electrophoresis to measure DNA strand breaks to assess the impact of 27HC on DNA damage. As seen in **Figures 4A**, **5A**, control LNCaP cells exhibited minimal DNA damage, as expected. However, DNA damage was increased several-fold by 27HC treatment. In LNCaP, adding cholesterol, but not androgens (R1881) blocked the DNA damaging effects of 27HC. The greatest effects were seen by the addition of olaparib, but the combination of olaparib + 27HC resulted in additive effects to further increase DNA damage. However, the effects of 27HC on DNA damage in DU145 cells were null except a minimal, but statistically significant, increase in DNA damage when 27HC was combined with olaparib from 1.0% of DNA in the tail to 2.5% (**Figures 4B**, **5B**).

## Discussion

Previous studies from our group showed that 27HC treatment lowered intracellular cholesterol and inhibited PC growth (14). Yet, the full mechanisms of 27HC-mediated anti-PC effects are not well understood. Specifically, we hypothesized that pathways beyond cholesterol inhibition are involved in the 27HC-mediated anti-PC effects that may interact with cholesterol inhibition. To test this, we performed a transcriptomic analysis of PC cells treated with 27HC or vehicle control to undercover novel mechanisms linking 27HC and PC growth. We found that 27HC treatment resulted in downregulation of multiple Hallmark pathways related to DNA damage repair, decreased expression of key cell cycle genes, induced “BRCAness”, increased markers of DNA damage and increased DNA damage that could be blocked by cholesterol add-back, but not androgen add-back. Results were much stronger in the AR-sensitive CYP27A1- cell line, LNCaP, vs. more modest effects in the AR-negative CYP27A1+ cell line, DU145. In human data, the link between CYP27A1 expression, the gene that encodes the enzyme that catalyzes 27HC synthesis, and DNA damage gene expression was corroborated and was strongest in high-grade CSPC, with a less clear association in mCPRC patients, suggesting our preclinical findings may accurately reflect human PC biology. Whether combining 27HC with treatments to further induce DNA damage (i.e., ionizing radiation) or approaches to limit DNA repair (i.e., PARP inhibitors) will result in synergistic anti-PC activities remains to be seen, though our data suggest these combinations may be fruitful, especially in high-grade CSPC.

After treatment with 27HC, gene expression analysis showed that both LNCaP cells and DU145 cells exhibited decreased expression of multiple DNA repair pathways in PC cells. 27HC-treated LNCaP cells specifically exhibited downregulation of pathways responsible for base excision repair, homologous recombination repair, mismatch repair, nucleotide excision repair, DNA replication and cell cycle. Further, 27HC treatment in LNCaP cells increased expression of markers of single-strand DNA breaks, suggesting 27HC increases DNA damage. Prior studies suggested that oxysterols beyond 27HC can induce DNA damage (31). For example, several oxysterols were shown to induce DNA damage and lead to apoptosis in the human cholangiocyte cell line MMNK-1 (32). It was suggested this may occur due to increased reactive oxygen species and lipid peroxides caused by the oxysterols vs. direct downregulation of DNA repair mechanisms, as seen in our study. Other oxysterols, such as 25HC and 7-ketocholesterol, can cause single-strand DNA breaks in human umbilical vascular epithelial cells and damage to full-length mitochondrial DNA, respectively (33, 34). Whether the mechanisms of this DNA damage are similar to the ones we identified or not remain to be tested. Nonetheless, our study, for the first time, showed that the oxysterol 27HC downregulates DNA repair pathway genes, increased canonical markers of DNA damage, and increased DNA damage as measured by the comet assay. Moreover, for the first time, we showed that this can occur in cancer cells, but did not occur in non-neoplastic prostate epithelial cells.

Both cell lines that we tested originated from patients with mCRPC, yet the LNCaP cell line responds more to the absence of androgens whereas DU145 cells are androgen insensitive (35). In contrast to robust responses in the AR-sensitive cell line LNCaP, results in the AR-negative DU145 cell line were more modest and mostly null. Specifically, in 27HC-treated DU145 cells, only DNA replication and base excision repair pathways were downregulated on gene expression analysis and expression of DNA damage markers was largely null. The response in AR-sensitive LNCaP cell lines to 27HC compared to AR-negative DU145 cells may suggest that 27HC has a more pronounced effect on DNA damage/repair pathway gene expression in androgen sensitive cancers. Alternatively, as LNCaP is CYP27A1- and DU145 is CYP27A1+, this may reflect the CYP27A1 status of the cells rather than androgen activity. Nonetheless, given that cholesterol is the precursor for androgens, this raises the possibility that the effects of 27HC are mediated, at least in part, via lowering androgens.

The link between AR and DNA damage repair has been well studied in preclinical models (36–38) and strong AR inhibition has been shown to create a synthetic lethality to PARP inhibitors (23). Recently, these preclinical observations were validated in a large phase 3 human clinical trial. Specifically, in the PROpel study, the PARP inhibitor, olaparib, in combination with the androgen-biosynthesis inhibitor abiraterone significantly improved imaging-based progression-free survival (ibPFS) in men with mCRPC compared to abiraterone plus placebo (39). This was true regardless of homologous recombination repair gene mutation (HRRm) status, though the benefits of combination therapy were greater in men with HRRm (hazard ratio 0.50, 95%CI 0.34-0.73) than men without HRRm (hazard ratio 0.76, 95%CI 0.60-0.97) (39). These results were further confirmed in the phase 3 TALAPRO-2 study, where mCRPC patients treated with the PARP inhibitor talazoparib plus the androgen receptor antagonists enzalutamide had significantly longer ibPFS then mCRPC patients treated with placebo plus enzalutamide (hazard ratio 0.63, 95%CI 0.51-0.78) (40). Again, ibPFS improvement was seen regardless of HRRm status, though again, ibPFS benefits were greater in men with HRRm (HR 0.46, 95%CI 0.30-0.70) than men without HRRm (HR 0.70, 95%CI 0.54-0.89). Nonetheless, the fact that even men with genomic HRR mutations had benefit supports the hypothesis that AR inhibition can create a sensitivity to PARP inhibitors given that PARP inhibitors as a single-agent in men without HRR mutations have no benefits (41).

Given the confirmation from phase 3 trials that strong androgen inhibition can induce sensitivity to PARP inhibitors, whether the observed findings in our study can be solely attributed to the known link between the AR and DNA damage response, considering the role of cholesterol as a precursor for androgens, remains a question. However, the fact that effects on DNA repair gene expression by microarray, albeit weaker, were seen in the AR-negative cell line DU145 and cholesterol add-back but not androgen add-back blocked the DNA damaging effects of 27HC in LNCaP cells (as shown by the comet assay), strongly suggests mechanisms beyond AR – specifically cholesterol mediated actions. Moreover, in LNCaP cells, while 27HC inhibited many pathways, decreased androgen signaling was not one of the top 10 pathways altered. As such, this lends further support that our findings cannot solely be explained by the known AR-DNA damage response link.

After further validation in other models varying in both AR and CYP27A1 status and using CYP27A1 overexpressing cells, future directions should involve exploring the potential of cholesterol inhibition, via 27HC or other mechanisms, in combination with treatments aimed at directly inducing DNA damage (radiation) or treatments aimed at preventing DNA repair (PARP inhibitors). Moreover, further mechanistic efforts are needed to tease out the effect of cholesterol inhibition, AR inhibition, or both on impairing DNA damage and synergizing with PARP inhibitors. Importantly, our data suggest these efforts are likely to be most fruitful in patients with CSPC and perhaps in those whose tumors lack CYP27A1 expression. While trials are ongoing testing AR inhibition with PARP inhibitors in men with CSPC (Talapro-3, AMPLITUDE) (42, 43), we are unaware of any combination trials testing cholesterol inhibition with PARP inhibition.

Our study demonstrates several strengths that contribute to the robustness of the findings. First, we investigated two different PC cell lines, LNCaP and DU145, which allowed us to capture potential variations in the observed outcomes across different cellular contexts. Additionally, we utilized multiple CSPC and CRPC human datasets, providing a broader perspective and enhancing the generalizability of our results. Moreover, we employed both microarray analysis and qRT-PCR to measure DNA damage gene expression, ensuring a comprehensive assessment of DNA repair gene expression. Lastly, our study incorporated multiple markers of DNA damage, including a comet assay, which adds further reliability and strengthens the validity of the conclusions. Overall, these methodological strengths enhance the rigor of the study and contribute to a more comprehensive understanding of the relationship between the AR, DNA damage response, and cholesterol metabolism in PC.

While our study presents valuable insights, there are certain limitations that need to be acknowledged. First, we only examined one AR sensitive cell line, which restricts the generalizability of the findings. Including additional AR sensitive cell lines would have provided a more comprehensive understanding of the cholesterol-AR-DNA damage response link. Also, while the reduction of DNA damage pathway gene expressions suggests 27HC induced DNA damage by impairing DNA damage repair, in the absence of additional experiments, we cannot prove this, and thus further studies are needed to determine whether 27HC impairs DNA damage repair or if it causes DNA damage in and of itself. Also, validation of other genes beyond RAD51 and FEN1 suggestively downregulated as seen by the microarray data is needed. Moreover, although the findings raise interesting hypotheses regarding the potential interaction of cholesterol with PARP inhibitors or DNA damaging agents such as radiation therapy, these hypotheses still need to be empirically tested in future studies. Also, for the human data, the genomic status of the tumors was generally not known and thus whether associations between CYP27A1 and DNA repair differ in tumors with DNA repair gene mutations is unknown. Finally, while our data suggest that lowering intracellular cholesterol via 27HC down-regulated expression of DNA repair genes and led to DNA damage, whether 27HC is the ideal “drug” to move forward with in human clinical trials remains to be seen. However, we would argue that alternative agents such as statins, which effectively lower serum cholesterol, may have limited tissue penetration into the tumor and thus may not be ideal. Specifically, a prior study from our team showed that lowering circulating cholesterol, in some models, can lead to upregulation of LDL receptor and maintained if not even higher tumor cholesterol (13). As such, future work should include better understanding the exact mechanism of 27HC mediated effects, better teasing out the specific effects of cholesterol versus AR signaling, and developing the best clinical “drug” to target intracellular cholesterol. Addressing these limitations will lead to a more comprehensive understanding of the complex mechanisms underlying PC and potentially guide the development of more effective therapeutic combination strategies.

## Conclusions

The results from this study suggest 27HC treatment, possibly via lowering intracellular cholesterol, inhibits expression of DNA repair pathways, induces “BRCAness” through downregulation of HR gene expression, and leads to increased DNA damage. These effects were stronger in LNCaP, an AR-sensitive CYP27A1- cell line, with weak to minimal effects seen in DU145, an AR-negative CYP27A1+ cell line. In human data, there was a robust association between higher CYP27A1, the gene encoding the enzyme that creates 27HC, and decreased DNA damage gene expression, especially in CSPC. Additional studies are needed to directly test whether 27HC creates a synthetic lethality to PARP inhibitors and other DNA damaging agents, especially in high-grade CSPC.

## Data availability statement

The raw data supporting the conclusions of this article will be made available by the authors, without undue reservation.

## Ethics statement

Ethical approval was not required for the studies on humans in accordance with the local legislation and institutional requirements because only commercially available established cell lines were used.

## Author contributions

GG: study conception and design, writing, data collection, analysis and interpretation of results, draft manuscript preparation and execution. NF: study conception and design, writing, draft manuscript preparation and execution, analysis and interpretation of results. SaD: writing, draft manuscript preparation. JD: writing, draft manuscript preparation. RS: data collection, analysis and interpretation of results. SS: data collection, analysis and interpretation of results. SP: data collection, analysis and interpretation of results. ShD: draft manuscript preparation. IV: draft manuscript preparation. KK: data collection. HT: data collection, analysis and interpretation of results. SY: data collection, analysis and interpretation of results. Y-JL: data collection, analysis and interpretation of results. WY: data collection, analysis and interpretation of results. JB: analysis and interpretation of results, draft manuscript revision. MF: analysis and interpretation of results, draft manuscript revision. SF: study conception and design, writing, draft manuscript preparation and execution, analysis and interpretation of results. All authors contributed to the article and approved the submitted version.

## References

[1] Bethesda M. SEER cancer stat facts: prostate cancer. United States: National Cancer Institute (2023).

[2] Peisch SF Van Blarigan EL Chan JM Stampfer MJ Kenfield SA. Prostate cancer progression and mortality: a review of diet and lifestyle factors. World J Urol (2017) 35(6):867–74. doi: 10.1007/s00345-016-1914-3 PMC5472048 27518576

[3] Oczkowski M Dziendzikowska K Pasternak-Winiarska A Włodarek D Gromadzka-Ostrowska J. Dietary factors and prostate cancer development, progression, and reduction. Nutrients (2021) 13(2). doi: 10.3390/nu13020496 PMC7913227 33546190

[4] Swyer G. The cholesterol content of normal and enlarged prostates. Cancer Res (1942) 2(5):372–5.

[5] Chen M Zhang J Sampieri K Clohessy JG Mendez L Gonzalez-Billalabeita E. An aberrant SREBP-dependent lipogenic program promotes metastatic prostate cancer. Nat Genet (2018) 50(2):206–18. doi: 10.1038/s41588-017-0027-2 PMC6714980 29335545

[6] Platz EA Till C Goodman PJ Parnes HL Figg WD Albanes D. Men with low serum cholesterol have a lower risk of high-grade prostate cancer in the placebo arm of the prostate cancer prevention trial. Cancer epidemiol Biomarkers Prev (2009) 18(11):2807–13. doi: 10.1158/1055-9965.EPI-09-0472 PMC2877916 19887582

[7] Jamnagerwalla J Howard LE Allott EH Vidal AC Moreira DM Castro-Santamaria R. Serum cholesterol and risk of high-grade prostate cancer: results from the REDUCE study. Prostate Cancer Prostatic Dis (2018) 21(2):252–9. doi: 10.1038/s41391-017-0030-9 PMC6021229 29282360

[8] Schnoeller TJ Jentzmik F Schrader AJ Steinestel J. Influence of serum cholesterol level and statin treatment on prostate cancer aggressiveness. Oncotarget (2017) 8(29):47110. doi: 10.18632/oncotarget.16943 28445145 PMC5564548

[9] El-Kenawi A Dominguez-Viqueira W Liu M Awasthi S Abraham-Miranda J Keske A. Macrophage-derived cholesterol contributes to therapeutic resistance in prostate cancer. Cancer Res (2021) 81(21):5477–90. doi: 10.1158/0008-5472.CAN-20-4028 PMC8563406 34301759

[10] Bansal D Undela K D’Cruz S Schifano F. Statin use and risk of prostate cancer: a meta-analysis of observational studies. PLoS One (2012) 7(10):e46691. doi: 10.1371/journal.pone.0046691 23049713 PMC3462187

[11] Goldberg H Mohsin FK Saskin R Kulkarni GS Berlin A Kenk M. The suggested unique association between the various statin subgroups and prostate cancer. Eur Urol focus (2021) 7(3):537–45. doi: 10.1016/j.euf.2020.06.005 32620539

[12] Peltomaa A Raittinen P Talala K Taari K Tammela LTJ Auvinen A. Prostate cancer prognosis after initiation of androgen deprivation therapy among statin users. A population-based cohort study. Prostate Cancer Prostatic Dis (2021) 24(3):917–24. doi: 10.1038/s41391-021-00351-2 PMC8384625 33790420

[13] Masko EM Alfaqih MA Solomon KR Barry WT Newgard CB Muehlbauer MJ. Evidence for feedback regulation following cholesterol lowering therapy in a prostate cancer xenograft model. Prostate (2017) 77(5):446–57. doi: 10.1002/pros.23282 PMC5822711 27900797

[14] Alfaqih MA Nelson ER Liu W Safi R Jasper JS Macias E. CYP27A1 loss dysregulates cholesterol homeostasis in prostate cancerCYP27A1 loss is involved in prostate cancer progression. Cancer Res (2017) 77(7):1662–73. doi: 10.1158/0008-5472.CAN-16-2738 PMC5687884 28130224

[15] Dambal S Alfaqih M Sanders S Maravilla E Ramirez-Torres A Galvan GC. 27-hydroxycholesterol impairs plasma membrane lipid raft signaling as evidenced by inhibition of IL6–JAK–STAT3 signaling in prostate cancer cells27HC disrupts lipid raft IL6–JAK–STAT3 in prostate cancer. Mol Cancer Res (2020) 18(5):671–84. doi: 10.1158/1541-7786.MCR-19-0974 PMC7971119 32019810

[16] Bello D Webber MM Kleinman HK Wartinger DD Rhim JS. Androgen responsive adult human prostatic epithelial cell lines immortalized by human papillomavirus 18. Carcinogenesis (1997) 18(6):1215–23. doi: 10.1093/carcin/18.6.1215 9214605

[17] Lockstone HE. Exon array data analysis using Affymetrix power tools and R statistical software. Brief Bioinform (2011) 12(6):634–44. doi: 10.1093/bib/bbq086 PMC3220870 21498550

[18] Piccolo SR Sun Y Campbell JD Lenburg ME Bild AH Johnson WE. A single-sample microarray normalization method to facilitate personalized-medicine workflows. Genomics (2012) 100(6):337–44. doi: 10.1016/j.ygeno.2012.08.003 PMC3508193 22959562

[19] Storey JD. A direct approach to false discovery rates. J R Stat Society: Ser B (Statistical Methodology) (2002) 64(3):479–98. doi: 10.1111/1467-9868.00346

[20] Subramanian A Tamayo P Mootha VK Mukherjee S Ebert BL Gillette MA. Gene set enrichment analysis: a knowledge-based approach for interpreting genome-wide expression profiles. Proc Natl Acad Sci (2005) 102(43):15545–50. doi: 10.1073/pnas.0506580102 PMC1239896 16199517

[21] Liberzon A Birger C Thorvaldsdóttir H Ghandi M Mesirov JP Tamayo P. The molecular signatures database hallmark gene set collection. Cell systems (2015) 1(6):417–25. doi: 10.1016/j.cels.2015.12.004 PMC4707969 26771021

[22] Levine DM Haynor DR Castle JC Stepaniants SB Pellegrini M Mao M. Pathway and gene-set activation measurement from mRNA expression data: the tissue distribution of human pathways. Genome Biol (2006) 7(10):R93. doi: 10.1186/gb-2006-7-10-r93 17044931 PMC1794557

[23] Li L Karanika S Yang G Wang J Park S Broom BM. Androgen receptor inhibitor-induced “BRCAness” and PARP inhibition are synthetically lethal for castration-resistant prostate cancer. Sci Signal (2017) 10(480). doi: 10.1126/scisignal.aam7479 PMC5855082 28536297

[24] Wood RD Mitchell M Lindahl T. Human DNA repair genes. Mutat Res (2005) 577(1-2):275–83. doi: 10.1016/j.mrfmmm.2005.03.007 15922366

[25] Kensler KH Awasthi S Alshalalfa M Trock BJ Freedland SJ Freeman MR. Variation in molecularly defined prostate tumor subtypes by self-identified race. Eur Urol Open Sci (2022) 40:19–26. doi: 10.1016/j.euros.2022.03.014 35638091 PMC9142751

[26] Howard LE Zhang J Fishbane N De Hoedt AM Klaassen Z Spratt DE. Validation of a genomic classifier for prediction of metastasis and prostate cancer-specific mortality in African-American men following radical prostatectomy in an equal access healthcare setting. Prostate Cancer Prostatic Dis (2020) 23(3):419–28. doi: 10.1038/s41391-019-0197-3 31844180

[27] Abida W Cyrta J Heller G Prandi D Armenia J Coleman I. Genomic correlates of clinical outcome in advanced prostate cancer. Proc Natl Acad Sci USA (2019) 116(23):11428–36. doi: 10.1073/pnas.1902651116 PMC6561293 31061129

[28] Fenor de la Maza MD Chandran K Rekowski J Shui IM Gurel B Cross E. Immune biomarkers in metastatic castration-resistant prostate cancer. Eur Urol Oncol (2022) 5(6):659–67. doi: 10.1016/j.euo.2022.04.004 PMC7617991 35491356

[29] Gyori BM Venkatachalam G Thiagarajan PS Hsu D Clement MV. OpenComet: an automated tool for comet assay image analysis. Redox Biol (2014) 2:457–65. doi: 10.1016/j.redox.2013.12.020 PMC3949099 24624335

[30] Cicenas J Kalyan K Sorokinas A Stankunas E Levy J Meskinyte I. Roscovitine in cancer and other diseases. Ann Trans Med (2015) 3(10). 10.3978/j.issn.2305-5839.2015.03.61 PMC4486920 26207228

[31] Kloudova A Guengerich FP Soucek P. The role of oxysterols in human cancer. Trends Endocrinol Metab (2017) 28(7):485–96. doi: 10.1016/j.tem.2017.03.002 PMC5474130 28410994

[32] Jusakul A Loilome W Namwat N Haigh WG Kuver R Dechakhamphu S. Liver fluke-induced hepatic oxysterols stimulate DNA damage and apoptosis in cultured human cholangiocytes. Mutat Res (2012) 731(1-2):48–57. doi: 10.1016/j.mrfmmm.2011.10.009 22044627

[33] Woźniak E Broncel M Bukowska B Gorzelak-Pabiś P. The protective effect of dabigatran and rivaroxaban on DNA oxidative changes in a model of vascular endothelial damage with oxidized cholesterol. Int J Mol Sci (2020) 21(6). doi: 10.3390/ijms21061953 PMC7139915 32182973

[34] Gramajo AL Zacharias LC Neekhra A Luthra S Atilano SR Chwa M. Mitochondrial DNA damage induced by 7-ketocholesterol in human retinal pigment epithelial cells in vitro. Invest Ophthalmol Vis Sci (2010) 51(2):1164–70. doi: 10.1167/iovs.09-3443 19834037

[35] Webber MM Bello D Quader S. Immortalized and tumorigenic adult human prostatic epithelial cell lines: characteristics and applications Part 2. Tumorigenic cell lines. Prostate (1997) 30(1):58–64. doi: 10.1002/(sici)1097-0045(19970101)30:1<58::aid-pros9>3.0.co;2-h 9018337

[36] Ta HQ Gioeli D. The convergence of DNA damage checkpoint pathways and androgen receptor signaling in prostate cancer. Endocrine-related cancer (2014) 21(5):R395. doi: 10.1530/ERC-14-0217 25096064 PMC4382101

[37] Luo H Liu Y Li Y Zhang C Yu B Shao C. Androgen receptor splicing variant 7 (ARv7) promotes DNA damage response in prostate cancer cells. FASEB J (2022) 36(9):e22495. doi: 10.1096/fj.202200190R 35947121

[38] Thompson TC Li L. Connecting androgen receptor signaling and the DNA damage response: Development of new therapies for advanced prostate cancer. Mol Cell Oncol (2017) 4(4):e1321167. doi: 10.1080/23723556.2017.1321167 28819638 PMC5540204

[39] Clarke NW Armstrong AJ Thiery-Vuillemin A Oya M Shore N Loredo E. Abiraterone and olaparib for metastatic castration-resistant prostate cancer. NEJM Evidence (2022) 1(9):EVIDoa2200043. doi: 10.1056/EVIDoa2200043 38319800

[40] Agarwal N Azad A Carles J Fay AP Matsubara N Heinrich D. TALAPRO-2: Phase 3 study of talazoparib (TALA)+ enzalutamide (ENZA) versus placebo (PBO)+ ENZA as first-line (1L) treatment in patients (pts) with metastatic castration-resistant prostate cancer (mCRPC). Am Soc Clin Oncol (2023). doi: 10.1200/JCO.2023.41.6_suppl.LBA17

[41] Mateo J Carreira S Sandhu S Miranda S Mossop H Perez-Lopez R. DNA-repair defects and olaparib in metastatic prostate cancer. N Engl J Med (2015) 373(18):1697–708. doi: 10.1056/NEJMoa1506859 PMC5228595 26510020

[42] Vis AN Noordzij MA Fitoz K Wildhagen MF Schroder FH van der Kwast TH. Prognostic value of cell cycle proteins p27(kip1) and MIB-1, and the cell adhesion protein CD44s in surgically treated patients with prostate cancer. J urol (2000) 164(6):2156–61. doi: 10.1016/S0022-5347(05)66989-3 11061947

[43] Thomas GV Schrage MI Rosenfelt L Kim JH Salur G deKernion JB. Preoperative prostate needle biopsy p27 correlates with subsequent radical prostatectomy p27, Gleason grade and pathological stage. J urol (2000) 164(6):1987–91. doi: 10.1016/S0022-5347(05)66934-0 11061897

