# Supplementary figures and images for: 27-hydroxycholesterol and DNA damage repair: implication in prostate cancer
Source: Front Oncol. 2023 Dec 21;13:1251297. doi: 10.3389/fonc.2023.1251297 (PMC10771304; doi:10.3389/fonc.2023.1251297)

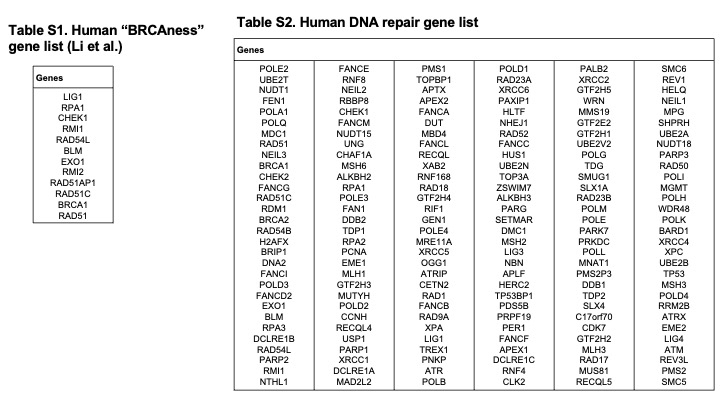

Supplement: Supplementary file 1 [file Image_1.jpeg]

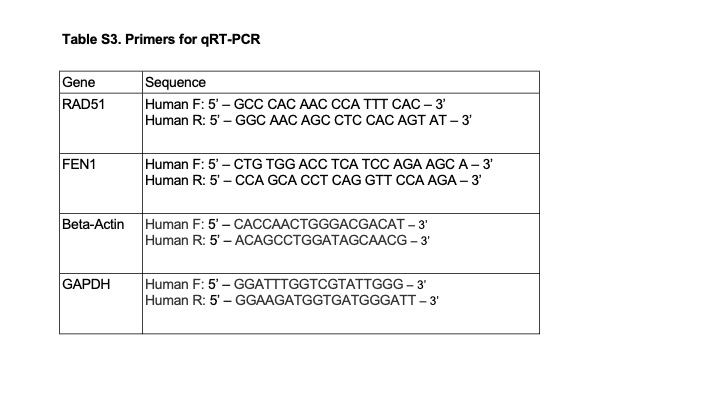

Supplement: Supplementary file 2 [file Image_2.jpeg]
